# Association between triglyceride glucose index (TyG) and psychotic symptoms in patients with first-episode drug-naïve major depressive disorder

**DOI:** 10.3389/fpsyt.2024.1342933

**Published:** 2024-02-23

**Authors:** Junjun Liu, Yangchun Wang, Wei Mu, Yang Liu, Ruixiang Tong, Zhaomin Lu, Hsinsung Yuan, Fengnan Jia, Xiaobin Zhang, Zhe Li, Wanqiu Yang, Xiangdong Du, Xiangyang Zhang

**Affiliations:** ^1^ Soochow University, Suzhou, China; ^2^ Nanjing Meishan Hospital, Nanjing, China; ^3^ Suzhou Guangji Hospital, The Affiliated Guangji Hospital of Soochow University, Suzhou, China; ^4^ School of Ethnology and Sociology, Yunnan University, Kunming, China; ^5^ CAS Key Laboratory of Mental Health, Institute of Psychology, Chinese Academy of Sciences, Beijing, China

**Keywords:** major depressive disorder, psychotic symptoms, triglyceride glucose index, insulin resistance, type 2 diabetes mellitus

## Abstract

**Objective:**

Major depressive disorder (MDD) sufferers frequently have psychotic symptoms, yet the underlying triggers remain elusive. Prior research suggests a link between insulin resistance (IR) and increased occurrence of psychotic symptoms. Hence, this study sought to investigate the potential association between psychotic symptoms in Chinese patients experiencing their first-episode drug-naïve (FEDN) MDD and the triglyceride glucose (TyG) index, an alternative measure of insulin resistance (IR).

**Methods:**

Between September 2016 and December 2018, 1,718 FEDN MDD patients with an average age of 34.9 ± 12.4 years were recruited for this cross-sectional study at the First Hospital of Shanxi Medical University in China. The study collected clinical and demographic data and included assessments of anxiety, depression, and psychotic symptoms using the 14-item Hamilton Anxiety Rating Scale (HAMA), the 17-item Hamilton Depression Rating Scale (HAMD-17), and the positive subscales of the Positive and Negative Syndrome Scale (PANSS), respectively. Measurements of metabolic parameters, fasting blood glucose (FBG), and thyroid hormones were also gathered. To assess the correlation between the TyG index and the likelihood of psychotic symptoms, the study used multivariable binary logistic regression analysis. Additionally, two-segmented linear regression models were employed to investigate possible threshold effects in case non-linearity relationships were identified.

**Results:**

Among the patients, 9.95% (171 out of 1,718) exhibited psychotic symptoms. Multivariable logistic regression analysis showed a positive correlation between the TyG index and the likelihood of psychotic symptoms (OR = 2.12, 95% CI: 1.21-3.74, P = 0.01) after adjusting for confounding variables. Moreover, smoothed plots revealed a nonlinear relationship with the TyG index, revealing an inflection point at 8.42. Interestingly, no significant link was observed to the left of the inflection point (OR = 0.50, 95% CI: 0.04-6.64, P = 0.60), whereas beyond this point, a positive correlation emerged between the TyG index and psychotic symptoms (OR = 2.42, 95% CI: 1.31-4.48, P = 0.01). Particularly, a considerable 142% rise in the probability of experiencing psychotic symptoms was found with each incremental elevation in the TyG index.

**Conclusions:**

Understanding the non-linear link between the TyG index and the risk of psychotic symptoms in Chinese patients with FEDN MDD highlights the potential for targeted therapeutic approaches. By acknowledging the threshold effect observed, there is an opportunity to mitigate risk factors associated with IR-related psychiatric comorbidities through tailored interventions. These preliminary results stress the need for further longitudinal research to solidify these insights and contribute to more effective therapeutic strategies.

## 1 Background

Major depressive disorder (MDD), one of the most prevalent mental illnesses, is characterized by major depressive episodes and includes symptoms such as weight loss, weariness, insomnia, depressed mood, reduced interest or pleasure, and recurrent thoughts of suicide (1). MDD constitutes a notable societal and individual burden, involving substantial medical and financial costs. According to the World Health Organization, depression is predicted to be the primary cause of worldwide illness burden by 2030, overtaking cardiovascular and respiratory diseases, ranking third in terms of disability-adjusted life years (2). A recent comprehensive study suggests a global lifetime prevalence of MDD ranging from 2 to 21% (3). The National Comorbidity Replication Survey conducted in America using the Diagnostic and Statistical Manual of Mental Disorders (DSM)-IV criteria revealed a 12-month estimate of 6.6% and a lifetime prevalence of 16.2% for MDD (4). Particularly in mainland China, the estimated lifetime prevalence of depression was 3.3% overall, with rural individuals appearing to be more susceptible to depression than urban ones (5). The impact of MDD on patients’ lives and society at large was enormous, as evidenced by the correlations between depression and suicidality, increased work loss and disability, worse quality of life, and increased financial and familial burden (6). Despite its substantial impact, the psychopathological mechanisms of MDD remain poorly understood.

A severe subtype of MDD, known as MDD with psychotic characteristics or psychotic depression, is characterized by major depressive episodes accompanied by tactile, somatic, paranoid, auditory, and guilt-related hallucinations (7). This subtype is linked to poorer outcomes in comparison to individuals with MDD without psychotic symptoms, regarding depressive episodes, suicide, hospitalizations, financial support, and social functioning (8, 9). Psychotic symptoms are used as an assessment of MDD severity in both the DSM-V and the International Classification of Diseases (ICD)-10, the current worldwide categorization and diagnostic systems for mental diseases. Epidemiological studies indicate that the prevalence of psychotic depression can range from 0.4 to 0.6% in general populations and from 25.3 to 44.7% in patient groups suffering from major depressive illness (9–11). Recent systematic review and meta-analysis findings show that the presence of psychosis during a major depressive episode doubles the likelihood of lifetime and acute phase suicide attempts, while also increasing the risk of developing psychosis in subsequent episodes (12). These results substantiate the distinction between psychotic and nonpsychotic depression. According to earlier research, individuals with MDD experiencing psychotic symptoms also exhibited more severe depressive symptoms, a higher risk of suicidality, a worse course of the illness, a higher rate of relapse, a greater impairment in social function, and a worse response to depression treatment. These findings suggest that co-occurring psychotic and MDD symptoms are predictive of poor clinical outcomes (9, 13). There are significant differences in the clinical appearance, diagnosis, therapy, and prognosis of depression with and without psychotic symptoms (14). Nonetheless, psychotic symptoms in depression are often overlooked, receiving inadequate research and identification, underscoring the critical need for further investigation.

Insulin resistance (IR), also known as reduced insulin responsiveness, is a common marker of type 2 diabetes, hypertension, lipid metabolic issues, and even cardiovascular disease. Research has found that the TyG index, which was used to assess IR with 84.0% sensitivity and 45.0% specifcity, can act as a reliable and easy-to-use stand-in marker for IR (15). Compared to the Homeostatic Model Assessment of Insulin Resistance (HOMA-IR), the gold standard method to measure IR, the TyG index performed better in detecting patients with IR than the HOMA-IR index. Depression and insulin resistance-related diabetes have been linked to a number of observational studies conducted on the general population (16–18). The reciprocal association between type 2 diabetes mellitus (T2DM) and mental health disorders is consistently supported by epidemiological studies and meta-analyses (19). Depression worsens the already compromised health outcomes, amplifies micro- and macrovascular issues, and elevates death rates among individuals with T2DM. Simultaneously, T2DM has been associated with greater treatment resistance, chronicity, and the onset of more severe depressive symptoms (19). Brain regions involved in mood regulation and cognition, such as the hippocampus, amygdala, and nucleus accumbens, contain neurons and glia with insulin receptors (20). Within these areas, the intricate network of insulin signaling plays a major role in regulating neuroprotection, neurogenesis, neurotransmission, and synaptic plasticity. High body mass index (BMI) is associated with long-term psychiatric hospitalizations across an individual’s lifetime, an earlier onset of MDD, and the presence of comorbidities, according to the European Group for the Study of Resistant Depression (GSRD) (21). Moreover, emerging research suggests that patients with psychotic disorders may display varying degrees of insulin resistance in the early stages of the disorder, exhibiting greater fluctuations in glucose, insulin, and lipid parameters compared to the healthy control group (22). At this stage, cumulative effects of drugs and lifestyle factors are relatively limited. In light of these findings, researchers should promptly investigate the pathophysiology underlying this two-way connection.

In recent years, the triglyceride-glucose (TyG) index produced by adding fasting plasma glucose and triglycerides, has emerged as a novel indicator of IR (23). TyG index has been linked to depression, heart disease, and prognosis of sickness (24). For example, Shi et al. found that people with higher TyG index scores are more likely to have symptoms of depression. In addition to sadness, IR may increase the likelihood of psychotic symptoms (25). Consequently, we hypothesized that the TyG index could serve as a useful marker for detecting psychotic symptoms in MDD patients. However, to our knowledge, no study has investigated the connection between the TyG index and psychotic symptoms in MDD patients, especially in the Chinese community. Hence, this study aims to assess the association between the TyG index and psychotic symptoms in a large Chinese patient population with first-episode and drug-naive MDD (FEDN).

## 2 Materials and methods

### 2.1 Study design and participants

This study was carried out in compliance with the Declaration of Helsinki and authorized by the Institutional Review Board (IRB) of the First Clinical Medical College, Shanxi Medical University (No. 2016-Y27). Prior to participation, all enrolled patients were thoroughly informed about the trial and provided written consent.

A cross-sectional study was conducted from 2016 to 2018 at the First Hospital of Shanxi Medical University in Taiyuan, Shanxi Province, China, a general hospital setting. Patients with FEND MDD were recruited, and a structured, self-designed questionnaire was used to collect their general information and sociodemographic features. Healthcare professionals oversaw the administration of measurements and tests in the medical, psychological, and laboratory domains of the assessment. To encourage continued engagement and compliance, participants received compensation and a report on their medical outcomes.

The study included 1718 patients with FEDN MDD, consisting of 588 men and 1130 women, meeting the DSM IV-TR criteria. The inclusion criteria were as follows: individuals of Han Chinese ethnicity; 18–60 years old; first episode depressed symptoms at present; no history of antidepressant drug use; and a 17-item Hamilton Depression Rating Scale (HAMD-17) score ≥ 24. The exclusion criteria comprised the following: pregnancy or nursing (n = 10); other psychiatric disorders on Axis I rather than MDD (n = 15); alcohol or drug abuse (n = 9); other physical illnesses such as cancer, persistent infections, epilepsy, brain injury, or stroke (n = 9); acute clinical conditions making reliable interviews difficult; and other unspecified factors (n = 14). Ultimately, 78 patients were excluded from the study.

### 2.2 Sociodemographic characteristics and anthropometric data

Using a standardized questionnaire, the study team collected general information and sociodemographic details from the participants during the visit, including age, sex, education level, marital status, and length of sickness. Educational attainment was categorized into four groups: postgraduate, college, senior high school, and junior high school. Marital status was classified as either single, divorced, widowed, or married.

Following a resting period of at least 15 minutes in a seated position, the systolic and diastolic blood pressures (SBP and DBP, respectively) of the right arm were determined by averaging two readings taken with a standard mercury sphygmomanometer. Each patient submitted standard anthropometric measures of their weight in kilograms (kg) and height in meters (m) for calculating their BMI, using the formula: BMI = weight/(height)^2^.

### 2.3 Blood samples

Each patient provided a fasting venous blood sample, analyzed at the hospital’s laboratory center. On the same day, several blood biomarkers were measured, including free triiodothyronine (FT3), free thyroxine (FT4), thyroid-stimulating hormone (TSH), thyroid peroxidase antibody (TPOAb), anti-thyroglobulin (TgAb), high-density lipoprotein cholesterol (HDL-C), low-density lipoprotein cholesterol (LDL-C), triglycerides (TG), and total cholesterol (TC), and blood glucose (FBG). TG and FBG were used to calculate the TyG index using the following formula: TyG = Ln (FBG (mg/dL) × TG (mg/dL)/2) (26).

### 2.4 Clinical interview assessments

Psychotic symptoms were measured using the Positive Subscale of the Positive and Negative Syndrome Scale (PANSS). Every item was given a score on a 7-point Likert scale, where 1 represented absence and 7 represented great severity. As a result, the PANSS positive subscale’s overall score varied from 7 to 49. For this study, psychotic symptoms were defined as patients scoring 15 or higher on the total positive subscale (27). Patients were divided into two groups based on whether they exhibited psychotic symptoms: the group with psychotic symptoms (n = 171) and the group without psychotic symptoms (n = 1547).

The study employed the HAMD-17 to evaluate the degree of depression symptoms (28). The scale consists of nine items with scores ranging from 0 (none) to 2 (symptom-specific severity description) and eight items with scores on a 5-point scale from 0 (not present) to 4 (severe). A higher score on the HAMD-17, ranging from 0 to 52, indicated more severe depressed symptoms.

The intensity of anxiety symptoms was assessed using the Hamilton Anxiety Rating Scale (HAMA) (29). This 14-item scale was scored on a 5-point Likert scale, ranging from 0 for absence to 4 for severe symptoms. Higher scores indicated more severe anxiety symptoms, and the total score varied from 0 to 56.

These rating scales were trained to be used by two qualified psychiatrists with a minimum of five years of clinical experience. The repeated assessments using these scales exhibited a correlation value exceeding 0.8, showing strong inter-observer reliability.

### 2.5 Statistical analysis

Using the 1-sample Kolmogorov-Smirnov test, the normal distribution of all continuous variables was examined. For non-normally distributed continuous variables, the median and interquartile range (IQR) were shown. Otherwise, the mean and SD were presented. Frequencies and percentages were used to display categorical data. Differences between various TyG tertile groups were analyzed by the One-Way ANOVA test (normal distribution), the Kruskal-Wallis H test (skewed distribution), or the chi-squared test (categorical variables). TyG index was investigated as both continuous and categorical variables based on tertiles, and a linear connection between the index and psychotic symptoms was estimated through logistic regression models. Regression model findings were displayed as unadjusted and adjusted odds ratios (ORs) with 95% confidence intervals (CIs). TyG index was converted to a categorical variable (tertile of TyG index) for sensitivity analysis, and the P value (P) for the trend of TyG index with categorical variables confirmed the outcome of TyG index as a continuous variable in the various models. To assess multicollinearity between independent variables, a variance inflation factor (VIF) was employed. A VIF > 5 indicated multicollinearity, which was not considered in the final model. The inclusion of potential confounders in the final multivariable model depended on their impact on the TyG index estimations concerning psychotic symptoms: affecting estimates by more than 10% or having a p < 0.10 in univariable analysis (30). The TyG index and psychotic symptoms were correlated using three different logistic regression models: an unadjusted model, Model I adjusted for age and sex, and Model II adjusted for age, sex, education, length of illness, HAMD, HAMA, BMI, SBP, DBP, TC, HDL-c, LDL-c, TSH, TgAb and TPOAb. If a non-linear relation between the TyG index and psychotic symptoms was detected, the threshold impact of TyG index on psychotic symptoms was estimated using a smoothing plot and a two-piecewise linear regression model based on the Generalized Estimating Equation (GEE).

The statistical software packages R (http://www.r-project.org, The R Foundation) and EmpowerStats (http://www.empowerstats.com, X&Y Solution, Inc., Boston, Massachusetts, USA) were used for all the studies. For a two-tailed test, a P < 0.05 was considered statistically significant.

## 3 Results

### 3.1 Baseline characteristics of participants

In total, 1718 FEDN MDD patients were included in this study. The average age of the participants was 34.87 ± 12.43 years, with a gender distribution of 34.23% male (588/1718) and 65.77% female (1130/1718). Among all participants, 171 patients (9.95%) exhibited symptoms of psychosis. The baseline participant characteristics are listed in **Table 1** based on the tertiles of the TyG index. Significant relationships were observed between TyG index tertiles and several variables (P < 0.05), such as psychotic symptoms, BMI, HAMD, HAMA, HDL-c, LDL-c, TGAb, TC, TG, and SBP.

**Table 1:** Baseline characteristics of participants.

Variables	Total	TyG index tertile			P-value
		T1 (6.64-8.80)	T2 (8.81-9.27)	T3 (9.28-10.44)	
N	1718	573	572	573	
Age (years)	34.87 ± 12.43	34.9 ± 12.5	34.6 ± 12.2	35.2 ± 12.6	0.736
Duration of illness (months)	6.3 ± 4.7	6.2 ± 4.8	6.6 ± 4.9	6.2 ± 4.5	0.325
Gender					0.131
Male	588 (34.23%)	196 (34.2%)	212 (37.1%)	180 (31.4%)	
Female	1130 (65.77%)	377 (65.8%)	360 (62.9%)	393 (68.6%)	
Education					0.513
Junior high school	413 (24.04%)	135 (23.6%)	128 (22.4%)	150 (26.2%)	
Senior high school	760 (44.24%)	243 (42.4%)	265 (46.3%)	252 (44.0%)	
College	449 (26.14%)	160 (27.9%)	151 (26.4%)	138 (24.1%)	
Postgraduate	96 (5.59%)	35 (6.1%)	28 (4.9%)	33 (5.8%)	
Marital status					0.603
Single	502 (29.22%)	162 (28.3%)	176 (30.8%)	164 (28.6%)	
Married	1216 (70.78%)	411 (71.7%)	396 (69.2%)	409 (71.4%)	
PANSS positive score	8.85 ± 4.40	8.07 ± 3.46	8.76 ± 4.34	9.72 ± 5.09	< 0.001
Psychotic symptoms					< 0.001
No	1547 (90.05%)	541 (94.4%)	517 (90.4%)	489 (85.3%)	
Yes	171 (9.95%)	32 (5.6%)	55 (9.6%)	84 (14.7%)	
HAMD	30.30 ± 2.94	29.5 ± 2.8	30.4 ± 2.9	31.0 ± 3.0	< 0.001
HAMA	20.80 ± 3.47	20.3 ± 3.3	20.7 ± 3.4	21.4 ± 3.6	< 0.001
BMI (kg/m^2^)	24.37 ± 1.92	24.2 ± 2.0	24.4 ± 1.9	24.5 ± 1.9	0.011
Systolic blood pressure (mmHg)	119.48 ± 10.91	118.1 ± 10.7	119.2 ± 10.8	121.1 ± 11.0	< 0.001
Diastolic blood pressure (mmHg)	75.95 ± 6.74	75.4 ± 6.7	75.8 ± 6.5	76.7 ± 7.0	0.004
FBG (mmol/l)	5.40 ± 0.65	5.2 ± 0.6	5.4 ± 0.6	5.6 ± 0.7	< 0.001
TC (mmol/l)	5.25 ± 1.11	4.9 ± 1.0	5.3 ± 1.0	5.6 ± 1.1	< 0.001
TG (mmol/l)	2.17 ± 0.99	1.2 ± 0.3	2.0 ± 0.4	3.2 ± 0.8	< 0.001
HDL-c (mmol/l)	1.22 ± 0.29	1.3 ± 0.3	1.2 ± 0.3	1.2 ± 0.3	< 0.001
LDL-c (mmol/l)	2.98 ± 0.86	2.9 ± 0.8	3.0 ± 0.8	3.0 ± 0.9	0.013
TSH (uIU/ml)	5.07 ± 2.56	4.4 ± 2.4	5.0 ± 2.4	5.8 ± 2.7	< 0.001
FT3 (pmol/l)	4.90 ± 0.72	4.9 ± 0.7	4.9 ± 0.7	4.9 ± 0.7	0.268
FT4 (pmol/l)	16.70 ± 3.10	16.8 ± 3.3	16.6 ± 3.0	16.7 ± 3.1	0.534
TGAb (IU/l)	21.46 (14.43-43.62)	21.10 (14.48-39.83)	20.47 (13.98-38.02)	22.48 (15.19-56.12)	0.017
TPOAb (IU/l)	17.43 (12.32-34.61)	18.38 (12.57-32.32)	16.54 (12.22-32.29)	18.40 (12.24-39.79)	0.265

The variables are presented as n (%) or the mean ± SD or median (quartile 1-quartile 3); TyG, triglyceride glucose; HAMD, 17-item Hamilton Rating Scale for Depression; HAMA, 14-item Hamilton Anxiety Rating Scale; BMI, body mass index; FBG, fasting blood glucose; TC, total cholesterol; TG, triglyceride; HDL-c, high-density lipoprotein cholesterol; LDL-c, low-density lipoprotein cholesterol; TSH, thyroid-stimulating hormone; FT3, free triiodothyronine; FT4, free thyroxine; TGAb, thyroglobulin antibody; TPOAb, thyroid peroxidase antibody.

### 3.2 Univariate analysis of psychotic symptoms

The univariate analysis found significant positive correlations (P < 0.05) between psychotic symptoms and the following parameters: age (OR = 1.01, 95% CI: 1.00, 1.03), HAMD (OR = 1.76, 95% CI: 1.62, 1.90), HAMA (OR = 1.91, 95% CI: 1.77, 2.08), BMI (OR = 1.09, 95% CI: 1.01, 1.18), SBP (OR = 1.05, 95% CI: 1.04, 1.07), DBP (OR = 1.07, 95% CI: 1.05, 1.10), FBG (OR = 2.05, 95% CI: 1.63, 2.58), TC (OR = 1.57, 95% CI: 1.36, 1.81), TG (OR = 1.34, 95% CI: 1.16, 1.54), LDL-c (OR = 1.41, 95% CI: 1.18, 1.69), TSH (OR = 1.52, 95% CI: 1.42, 1.63), TGAb (OR = 1.001, 95% CI: 1.001, 1.002), TPOAb (OR = 1.002, 95% CI: 1.001, 1.003), TyG index (OR = 2.66, 95% CI: 1.88, 3.78), TyG tertile T2 (OR = 1.80, 95% CI: 1.14, 2.83 vs. T1) and TyG tertile T3 (OR = 2.90, 95% CI: 1.90, 4.44 vs. T1). Conversely, senior high school (OR = 0.60, 95% CI: 0.41, 0.87 vs. junior high school), college (OR = 0.51, 95% CI: 0.25, 1.18 vs. junior high school), and HDL-c (OR = 0.39, 95% CI: 0.23, 0.69) were all negatively correlated with psychotic symptoms (P < 0.05). The univariate analysis results are shown in **Table 2**.

**Table 2:** Univariate analysis for psychotic symptoms in patients with FEDN MDD.

Covariate	OR	95% CI	P-value
N	1718		
Age (years)	1.01	1.00, 1.03	0.036
Duration of illness (months)	1.02	0.99, 1.05	0.286
Gender			
Male	1.0 (Refrence)		
Female	1.42	1.00, 2.02	0.051
Education			
Junior high school	1.0 (Refrence)		
Senior high school	0.60	0.41, 0.87	0.007
College	0.51	0.33, 0.79	0.003
Postgraduate	0.55	0.25, 1.18	0.125
Marital status			
Single	1.0 (Refrence)		
Married	1.03	0.73, 1.46	0.864
HAMD	1.76	1.62, 1.90	< 0.001
HAMA	1.91	1.77, 2.08	< 0.001
BMI (kg/m^2^)	1.09	1.01, 1.18	0.037
Systolic blood pressure (mmHg)	1.05	1.04, 1.07	< 0.001
Diastolic blood pressure (mmHg)	1.07	1.05, 1.10	< 0.001
FBG (mmol/l)	2.05	1.63, 2.58	< 0.001
TC (mmol/l)	1.57	1.36, 1.81	< 0.001
TG (mmol/l)	1.34	1.16, 1.54	< 0.001
HDL-c (mmol/l)	0.39	0.23, 0.69	0.001
LDL-c (mmol/l)	1.41	1.18, 1.69	< 0.001
TSH (uIU/ml)	1.52	1.42, 1.63	< 0.001
FT3 (pmol/l)	0.98	0.79, 1.22	0.841
FT4 (pmol/l)	1.00	0.95, 1.05	0.929
TGAb (IU/l)	1.00	1.00, 1.00	< 0.001
TPOAb (IU/l)	1.00	1.00, 1.00	< 0.001
TyG index	2.66	1.88, 3.78	< 0.001
TyG tertile			
T1	1.0 (Refrence)		
T2	1.80	1.14, 2.83	0.011
T3	2.90	1.90, 4.44	< 0.001

HAMD, 17-item Hamilton Rating Scale for Depression; HAMA, 14-item Hamilton Anxiety Rating Scale; BMI, body mass index; FBG, fasting blood glucose; TC, total cholesterol; TG, triglyceride; HDL-c, high-density lipoprotein cholesterol; LDL-c, low-density lipoprotein cholesterol; TSH, thyroid-stimulating hormone; FT3, free triiodothyronine; FT4, free thyroxine; TGAb, thyroglobulin antibody; TPOAb, thyroid peroxidase antibody.

### 3.3 Associations between TyG index and psychotic symptoms

After fully adjusting the data, results showed a strong correlation between a higher TyG score and a higher likelihood of psychotic symptoms (**Table 3**; OR = 2.12, 95% CI: 1.21 to 3.74, P = 0.01). Particularly, patients in the third tertile of the TyG index demonstrated a higher probability of exhibiting psychotic symptoms (OR = 2.34, 95% CI: 1.20 to 4.75, P = 0.01) when compared to those in the first tertile.

**Table 3:** The results of two-piecewise Logistic regression model.

Inflection point of TyG index	OR	95%CI	*P*-value
Inflection point	8.42	8.28 to 8.58	
< 8.42 slope 1 (n=151)	0.50	0.04 to 6.64	0.60
≥ 8.42 slope 2 (n=1,567)	2.42	1.31 to 4.48	0.01
slope 2 – slope 1	4.87	1.28 to 18.46	0.02
predicted at 9.29	-2.94	-3.30 to -2.60	
Log likelihood ratio test			0.03

Effect: Psychotic symptoms; Cause: TyG index; adjusted for age, sex, education, duration of illness, HAMD, HAMA, BMI, SBP, DBP, TC, HDL-c, LDL-c, TSH, TGAb, TPOAb.

Slope 1 and slope 2 are the slope coefficients for the segment before and after the inflection point.

### 3.4 The non-linear relationship between TyG index and psychotic symptoms

**Figure 1:**
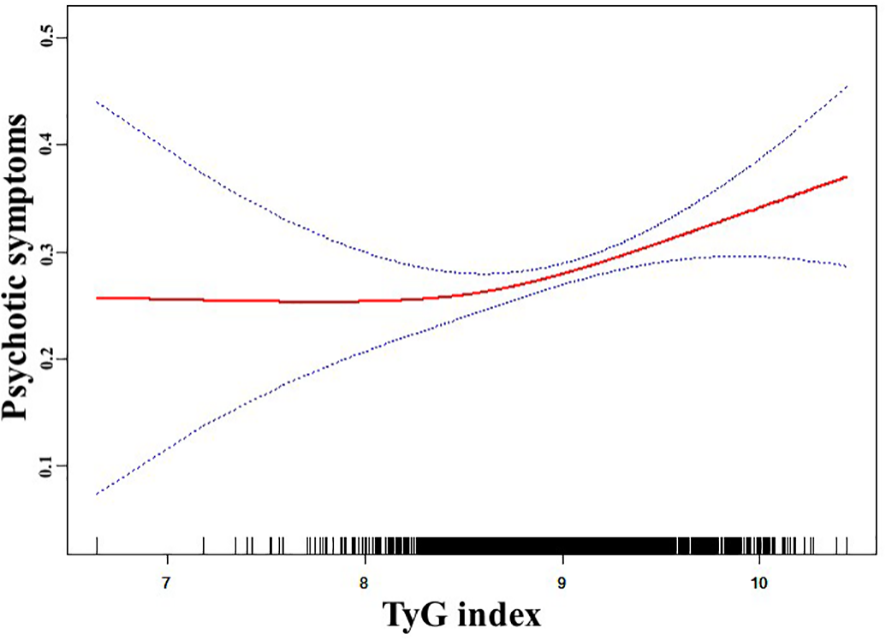
The relationship between TyG index and psychotic symptoms. A nonlinear relationship between TyG index and the probability of psychotic symptoms was observed after adjusting for age, sex, education, duration of illness, HAMD, HAMA, BMI, SBP, DBP, TC, HDL-c, LDL-c, TSH, TGAb, and TPOAb (P for non-linearity <0.05).

Using generalized additive models, **Figure 1** depicts the non-linear relationship (P for non-linearity < 0.05) between the TyG score and psychotic symptoms. A two-segment logistic regression model identified an inflection point value of 8.42 for the TyG index. There were 151 patients with a TyG index >= 8.42 and 1567 patients with a TyG index < 8.42. The chance of psychotic symptoms increased significantly by 142% for every unit increase in the TyG index on the right side of the inflection point (OR = 2.42, 95% CI: 1.31 to 4.48, P = 0.01). **Table 4** illustrates that there was no indication of a significant association between the TyG index and psychotic symptoms on the left side of the inflection point (OR = 0.50, 95% CI: 0.04 to 6.64, P = 0.60). The determination of the 95% CIs around the TyG index inflection point of 8.28 to 8.58 was achieved using bootstrap methods.

**Table 4:** Relationship between TyG index and psychotic symptoms in different models.

Variable	N	Unadjusted Model		Model I		Model II	
		OR (95%CI)	*P*-value	OR (95%CI)	*P*-value	OR (95%CI)	*P*-value
TyG index	1718	2.66 (1.88, 3.78)	< 0.001	2.63 (1.85, 3.74)	< 0.001	2.12 (1.21, 3.74)	0.01
TyG index tertile							
T1 (6.64-8.80)	573	Refrence		Refrence		Refrence	
T2 (8.81-9.27)	572	1.80 (1.14, 2.83)	0.01	1.83 (1.16, 2.87)	0.01	1.85 (0.96, 3.59)	0.07
T3 (9.28-10.44)	573	2.90 (1.90, 4.44)	< 0.001	2.88 (1.88, 4.41)	< 0.001	2.34 (1.20, 4.57)	0.01
*P* for trend		< 0.001		< 0.001		0.02	

OR, odds ratio; CI, confidence interval; Unadjusted Model adjusted for none; Model I adjusted for age, sex; Model II adjusted for age, sex, education, duration of illness, HAMD, HAMA, BMI, SBP, DBP, TC, HDL-c, LDL-c, TSH, TGAb, TPOAb.

## 4 Discussion

To our knowledge, this study marks the first investigation in China exploring the connection between psychotic symptoms and the TyG index within a reasonably large sample of individuals diagnosed with FEDN MDD. Our results demonstrate that the TyG index and psychotic symptoms are strongly correlated, even after adjusting for potential confounding variables. Moreover, there was a non-linear relationship between the TyG index and psychotic symptoms, with an inflection point of 8.42. On the right side of the inflection point, there was a significant 142% increase in the likelihood of psychotic symptoms for every unit increase in TyG index, yet no significant association was found on the left side of the inflection point. These findings shed new light on the relationship between the TyG index and psychotic symptoms in MDD patients. However, a comprehensive understanding of the underlying mechanisms necessitates further research, considering that alternative hypotheses may exist.

In our study, the prevalence of psychotic symptoms in MDD patients was found to be 9.95%, a lower prevalence compared to the range of 27 to 42% reported in most prior studies (11, 13, 31, 32), with only one indicating a prevalence of 5.3% (9). Numerous reasons could be contributing to this disparity. First, differences in psychiatric illness prevalence across studies could stem from the use of different diagnostic approaches (33). Second, the majority of the study participants in earlier investigations were long-term patients who were inpatients, outpatients, and members of the community. Of note, psychotic symptoms are more common in MDD inpatients, reaching 42% in a most recent study (11), compared to 14 to 20% in MDD outpatients (10, 31). Third, our study focused on individuals experiencing their first episode without prior drug usage. It is conceivable that as MDD progresses, certain patients may experience psychotic symptoms during their illness, resulting in a higher prevalence of MDD in chronic patients. Lastly, factors such as sample size, age, ethnicity, and gender ratio might also impact prevalence rates. Psychiatric symptoms are among the most prevalent symptoms in MDD patients, and while previous studies in various populations have shown varying prevalence of psychiatric symptoms, nearly all of them have shown that psychiatric symptoms require special attention in their management and treatment.

Clinically, insulin resistance refers to the inability of a known amount of endogenous or exogenous insulin to raise an individual’s absorption and utilization of glucose to the same extent as it does in the normal population (34). In our study, the TyG index, an indicator of insulin resistance, showed a positive correlation with psychotic symptoms, a correlation that persisted even after considering relevant confounding factors. In addition to being frequently connected to blood glucose problems in depressed individuals, IR has been related to psychotic symptoms. For example, a cross-sectional study conducted in South Korea involving over 160,000 participants discovered that IR was associated with a higher risk of depression, showing a 17 and 4% rise in incidence among young adults and non-diabetics, respectively (35). Moreover, it has been demonstrated that suicidal conduct in depression is associated with alterations in glucose metabolism and insulin resistance (36). Few studies have investigated the relationships between psychotic symptoms, insulin resistance, glucose levels, and depression. However, prior research indicates that dysthymia and recurrent or psychotic depression are related to higher glucose levels and higher HbA1c concentrations, respectively (37). Furthermore, interventions aimed at addressing depressive symptoms in adults with T2DM and/or significant depression have demonstrated improvements in indicators of insulin resistance or glucose impairment in adults, even without changes in body weight or adiposity (38).

The interaction between dyslipidemia and depression, leading to mental symptoms, primarily occurs through the inflammatory system. Multiple inflammatory cytokines have been robustly demonstrated to be positively correlated with depression and schizophrenia (39). Specifically, young adults with elevated IL-6 levels during childhood exhibit a higher likelihood of experiencing psychotic symptoms, while adolescents with increased C-reactive protein (CRP) levels tend to display subclinical psychotic symptoms (40). The trans-diagnostic impact of inflammation might be attributed to the correlation between CRP and symptoms that are common to both mood and psychotic illnesses, such as auditory hallucinations and anhedonia (40). Additionally, there was a positive correlation found between inflammatory markers (hs-CRP, MCP-1, TNF-alpha, and PAI-1 levels), lipid profiles (LDL-c, TG, and TC levels), and insulin resistance (fasting glucose level, fasting insulin level, and HOMA-IR) (41). These interactions may potentially exacerbate psychotic symptoms in MDD patients. Moreover, brain anatomy can be impacted by blood lipids, as higher TC levels cause a reduction in hippocampal volume (42). Of note, a recent study found that patients with bipolar disorder and higher levels of lipids displayed smaller brain structures than healthy controls (42). Additionally, it has been observed that compared to patients with less severe depression, those with psychotic depression had decreased hippocampus sizes (43). Consequently, the development of psychotic symptoms could potentially be mediated by TC-induced alterations in brain volume. In our study, statistical significance between the TyG index and psychotic symptoms in MDD was only found when TyG index was higher than 8.42, suggesting a threshold effect. Other recent research also disclosed a threshold impact with a turning point value of 9.29 between the TyG index and suicide attempts in MDD patients (44). While the threshold inflection points differ between these studies, they collectively indicate that exceeding the TyG index threshold significantly increases the risk of psychiatric symptoms and suicide attempts. As mentioned above, prior research has established links between psychiatric symptoms and inflammatory cytokines, which in turn are associated with lipid profiles. In turn, these lipid profiles have been correlated with brain anatomical anomalies. With the confirmed positive correlation between the TyG index and psychiatric symptoms, as shown in this study, it becomes crucial to comprehensively understand the intricate interplay among these factors to unravel the underlying pathological mechanisms.

The prevalence of MDD and conditions associated with IR, such as obesity, T2DM, and metabolic syndrome, is rapidly escalating and stands as the primary cause of disability in the world (45). Multiple epidemiological studies have demonstrated that individuals with MDD have an increased risk of IR-related disorders, with up to a 4-fold increased risk of MDD in those affected by T2DM and obesity (45). Additionally, a reduction in life expectancy of 10 to 20 years has been connected to MDD (46), where somatic disorders related to IR could be a contributing factor. Moreover, IR-related comorbidities in MDD have been linked to greater depression intensity, chronicity, and less favorable treatment outcomes (47, 48). The coexistence of depression in T2DM patients escalates mortality risk by 54%. In addition to environmental risk factors, genomic research has revealed a polygenic risk that is shared by disorders connected to IR and MDD, implying overlapping etiopathological pathways (49). Of note, dysregulations of immunological inflammation, gut microbiota, brain insulin signaling, and the hypothalamic-pituitary-adrenal axis are among the hypothesized common causes (50). Specifically, insulin signaling influences neurotransmission, synaptic plasticity, neuroprotection, and neurogenesis, with its receptors expressed in both neurons and glia in brain regions related to mood regulation and cognition, including the hippocampus, amygdala, and nucleus accumbens (20). Indeed, an altered brain insulin signaling has been described to impact the dopaminergic mesolimbic circuit (51), a significant pathway in the limbic system where dopamine acts as a major neurotransmitter. In addition, insulin levels were found to influence dopamine and other crucial neurotransmitters related to the phenomenology and management of major depressive disorder (52). For instance, insulin-resistant animal models show increased dopamine concentration in limbic and striatal regions, indicating a potential role for insulin resistance in the initiation or maintenance of psychotic symptoms through the enhancement of mesolimbic dopaminergic transmission (53).

Nonetheless, there has been some evidence that certain antidepressant and antidiabetic medications can improve insulin sensitivity measures or alleviate depressive symptoms. Particularly, selective serotonin reuptake inhibitors (SSRIs), the most commonly prescribed antidepressants, have been shown in short-term studies to improve glycemic control in individuals with comorbid MDD and T2DM, with no negative long-term effects on glycemic homeostasis (50). Due to these positive effects, SSRIs are typically regarded as first-line treatments for those with concomitant IR. In humans, the norepinephrine-dopamine reuptake inhibitor bupropion induces weight loss by modifying the mesolimbic reward circuit. In rats subjected to a high-fat diet, bupropion improved insulin sensitivity and glycolipid indicators (54). Consequently, bupropion might be especially intriguing for people with comorbid MDD and IR. In contrast, monoamine oxidase inhibitors and tricyclic antidepressants cause weight gain and upset glycemic homeostasis, making them less suitable for patients with IR (55). Furthermore, several oral hypoglycemic agents including biguanides (e.g., metformin), peroxisome proliferator-activated receptor-γ (PPAR- γ) agonists (e.g., rosiglitazone, pioglitazone), and glucagon-like peptide 1 receptor agonists (GLP1RA; e.g., liraglutide) have been tested as potential adjunctive therapies for depression (56). Specifically, metformin was shown to not be more effective than controls in reducing depressive symptomatology in a recent meta-analysis (57).

To lower the population’s risk of IR and, consequently, the chance of depression and cardio-metabolic disorders, clinicians must work to promote healthier lifestyles. Physical exercise could help mitigate IR and constitutes an effective depression therapy (58). Adopting a Mediterranean diet has shown protective effects against insulin resistance and the onset of depression while alleviating depressive symptoms and enhancing cognitive function in individuals with T2DM (59). Furthermore, prebiotics play a role in supporting cognitive and antidepressant effects, as well as improving insulin sensitivity by maintaining the homeostasis of the gut microbiota (60, 61). Therefore, implementing more tolerable and tailored treatment approaches alongside promoting healthier lifestyles can serve as vital prevention interventions. Given their significant impact, physicians should prioritize these interventions to effectively reduce potential risk factors associated with both IR-related conditions and psychiatric multimorbidity.

The present investigation offers several advantages. Firstly, it eliminates the impact of confounding variables such as disease duration, lifestyle modifications, medication, and concomitant medical problems by focusing on a research group of individuals with FEDN MDD. Secondly, multivariable logistic regression analysis and smoothing plots enable exploration of both linear and nonlinear relationships between the TyG index and psychotic symptoms. However, certain limitations warrant attention. Namely, the cross-sectional nature of this study precludes it from establishing causation. Thus, employing longitudinal methods in future investigations can better explore the causal relationship between the TyG index and psychotic symptoms in MDD patients. Second, the study’s exclusive focus on Han Chinese patients recruited from a general hospital’s mental outpatient department in Taiyuan (Shanxi Province, China) emphasizes the need to validate the present findings in individuals with different clinical backgrounds and ethnicities. Third, the inclusion of MDD patients who had not sought previous therapy for their depressed symptoms and were experiencing their first episode does not exclude those whose diagnosis later shifted to bipolar disorder. Fourth, as the study focused on individuals with severe MDD (HAMA mean > 20, 17-item HAMD scores ≥ 24), its findings might not apply to individuals with milder pathology. Fifth, the HOMA-IR, or revised quantitative insulin sensitivity check index (QUICKI), is the most widely used surrogate measure of insulin resistance. It is calculated using fasting insulin and glucose measurements. Unfortunately, relevant indicators were not assessed in this study. Lastly, a variety of confounding factors, such as diet, physical activity, drinking, smoking, personality traits, family income, social standing, and biological factors, were not investigated and could have a significant impact on the relationship between the TyG index and psychotic symptoms. Future studies should include a wider array of confounding variables to better understand the pathophysiological mechanisms behind the association between the TyG index and psychotic symptoms in people with MDD.

In conclusion, our study identified a nonlinear relationship between the TyG index and psychotic symptoms in Chinese patients with FEDN MDD, with an inflection point at approximately 8.42. Notably, we observed that psychotic symptoms were positively correlated with TyG index on the right side of this inflection point, while no significant correlation surfaced on the left side. Specifically, there was a considerable 142% rise in the probability of experiencing psychotic symptoms with each incremental elevation in the TyG index. Due to the cross-sectional methodology, limited understanding of the underlying mechanisms, and other study limitations, these results constitute a valuable preliminary report. Subsequent research employing a longitudinal design and structured evaluation tools is imperative to fully validate these findings, enhance our understanding of this correlation, and offer valuable insights into potential therapeutic strategies.

## Data availability statement

The original contributions presented in the study are included in the article/supplementary material. Further inquiries can be directed to the corresponding authors.

## Ethics statement

The studies involving humans were approved by the Institutional Review Board of the First Hospital, Shanxi Medical University (No. 2016-Y27). The studies were conducted in accordance with the local legislation and institutional requirements. The participants provided their written informed consent to participate in this study. Written informed consent was obtained from the individual(s) for the publication of any potentially identifiable images or data included in this article.

## Author contributions

JL: Investigation, Methodology, Writing – original draft. YW: Investigation, Writing – original draft. WM: Investigation, Writing – review & editing. LY: Formal Analysis, Investigation, Writing – original draft. RT: Investigation, Methodology, Writing – original draft. ZLu: Conceptualization, Data curation, Investigation, Writing – original draft. HY: Investigation, Writing – original draft. FJ: Investigation, Methodology, Software, Writing – original draft. XBZ: Validation, Writing – review & editing. ZLi: Funding acquisition, Resources, Validation, Writing – original draft. WY: Funding acquisition, Investigation, Visualization, Writing – original draft. XD: Supervision, Validation, Writing – review & editing. XYZ: Supervision, Validation, Writing – review & editing.
